# Spatial Analysis of Pulmonary Tuberculosis in Antananarivo Madagascar: Tuberculosis-Related Knowledge, Attitude and Practice

**DOI:** 10.1371/journal.pone.0110471

**Published:** 2014-11-11

**Authors:** Sitraka Rakotosamimanana, Vatsiharizandry Mandrosovololona, Julio Rakotonirina, Joselyne Ramamonjisoa, Justin Rasolofomanana Ranjalahy, Rindra Vatosoa Randremanana, Fanjasoa Rakotomanana

**Affiliations:** 1 Département de géographie, Faculté des Lettres et Sciences humaines, Université d′ Antananarivo, Antananarivo, Madagascar; 2 Institut National de Santé Publique et Communautaire, Antananarivo, Madagascar; 3 Etablissement Universitaire de Soins de Santé Publique d′Analakely, Antananarivo, Madagascar; 4 Institut Pasteur, Antananarivo, Madagascar; Fundació Institut d’Investigació en Ciències de la Salut Germans Trias i Pujol. Universitat Autònoma de Barcelona. CIBERES, Spain

## Abstract

**Introduction:**

Tuberculosis infection may remain latent, but the disease is nevertheless a serious public health issue. Various epidemiological studies on pulmonary tuberculosis have considered the spatial component and taken it into account, revealing the tendency of this disease to cluster in particular locations. The aim was to assess the contribution of Knowledge Attitude and Practice (KAP) to the distribution of tuberculosis and to provide information for the improvement of the National Tuberculosis Program.

**Methods:**

We investigated the role of KAP to distribution patterns of pulmonary tuberculosis in Antananarivo. First, we performed spatial scanning of tuberculosis aggregation among permanent cases resident in Antananarivo Urban Township using the Kulldorff method, and then we carried out a quantitative study on KAP, involving TB patients. The KAP study in the population was based on qualitative methods with focus groups.

**Results:**

The disease still clusters in the same districts identified in the previous study. The principal cluster covered 22 neighborhoods. Most of them are part of the first district. A secondary cluster was found, involving 18 neighborhoods in the sixth district and two neighborhoods in the fifth. The relative risk was respectively 1.7 (p<10^−6^) in the principal cluster and 1.6 (p<10^−3^) in the secondary cluster. Our study showed that more was known about TB symptoms than about the duration of the disease or free treatment. Knowledge about TB was limited to that acquired at school or from relatives with TB. The attitude and practices of patients and the population in general indicated that there is still a stigma attached to tuberculosis.

**Conclusion:**

This type of survey can be conducted in remote zones where the tuberculosis-related KAP of the TB patients and the general population is less known or not documented; the findings could be used to adapt control measures to the local particularities.

## Introduction

Recently, multidisciplinary approaches have been developed to study the role of external factors, such as physical, chemical, climatic, socioeconomic and cultural relations, in the development of diseases. Some diseases have spatial and temporal characteristics, and spatial factors might play a role in structuring the diffusion and development of health phenomena [1], [2], [3]. Geographic Information Systems (GIS) combined with spatial statistical analysis methods have been applied in studying tuberculosis transmission [4], [5], [6]. Integrating and taking into account the spatial component in many studies on pulmonary tuberculosis (TB) has revealed the tendency of this disease to cluster in particular locations [5], [7]. It would be informative to understand the contribution of spatial factors to the transmission of tuberculosis in these clusters. The value of using spatial and space-time analyses to describe tuberculosis distribution and concentration in some locations has been demonstrated [8], [9]. Modeling risk and tuberculosis patterns is useful because it allows the identification of priority areas to target interventions; it also reveals risk factors associated with these clusters such that models of risk transmission can be developed.

Risk modeling has been performed for pulmonary tuberculosis in the city of Antananarivo in 2004–2006 and revealed spatial clusters in the first, third and fourth districts; the average incidence of TB was 74.7 per 100,000 inhabitants [10]. The first district is the most populous and the first and fourth are both vulnerable to the risk of flooding [11].

The sites of spatial aggregations were considered as areas at risk, and their identification allows the establishment of priorities for intervention, including active case detection, and improving sanitation, hygiene and nutritional status locally. Randremanana *et al.* found that risk factors were significantly increased in households with more than one case [10]. Ownership of televisions was shown to be a risk factor. Watching television in overcrowded household may increase the risk of TB transmission [10]. Maciel *et al.* found a significant relationship between TB incidence and socioeconomic status in Victoria, Brazil [12].

High population density may increase the risk of transmission, and socioeconomic conditions represent a risk factor for TB. Also, the KAP of populations may play an important role in facilitating or impeding the spread of the disease. The KAP in Madagascar as relevant to TB is not well documented. Here, we report a two–part study: one involved the detection of clusters of TB cases among permanent residents of Antananarivo; the second is an analysis of tuberculosis-related KAP among patients and the population more generally. The aim was to assess the contribution of KAP to the distribution of tuberculosis then to provide information for the improvement of the National Tuberculosis Program.

## Materials and Methods

### Description of the study area

Antananarivo consists of an urban core and 30 municipalities that form an agglomeration; it is the most populous city and capital of Madagascar with 1,300,000 inhabitants. The municipalities are joined into what is known as FIFTAMA (Farimbona Iombonan'ny Firaisan'ireo Tanana Manodidina an'Antananarivo), forming a suburban belt around the urban city. Antananarivo Urban Township covers 87 km^2^. It is divided into six districts and into 192 “*fokontany*” (neighborhoods). Antananarivo is also a cosmopolitan city, and includes various ethnic populations. Socioeconomic inequality exists both within neighborhoods and between them. Some neighborhoods are more disadvantaged than others, with heterogeneous incomes and diverse characteristics of the local environments. The most deprived neighborhoods are in areas prone to flooding and are densely populated and insalubrious: the inhabitants of these neighborhoods are exposed to recurrent problems, increasing their vulnerability to epidemics [11], [13], [14].

### Identification of spatial clusters

We report a retrospective study based on smear-positive new cases of tuberculosis, among the residents of Antananarivo. The patients were reported between July 2010 and 2011 in 18 Diagnosis and Treatment Centers (DCT). Three of the DCT are in suburban zones, and permanent residents of Antananarivo are registered at these centers. Patient information was collected from the tuberculosis notifications register: gender, age, type of tuberculosis (positive pulmonary smear, negative pulmonary smear, extra-pulmonary), phase of treatment, and patient status. The patients' addresses and other details were obtained from individual treatment cards; the addresses were verified at the “*fokontany*" administration office by the investigator.

The spatial aggregation of new tuberculosis cases was studied with the software SaTScan v9.1.1 Spatial and Space Time Scan Statistics. SaTScan software, the trademark belonging to Martin Kulldorff [15], was developed jointly by: (i) Martin Kulldorff, (ii) the national cancer institute, and (iii) Farzad Mostashari of the New York City Department of Health and Mental Hygiene. The distribution of new cases of tuberculosis in each neighborhood was considered to conform to a Poisson distribution. The null hypothesis was that the new cases of tuberculosis are randomly distributed throughout the neighborhoods of the urban districts of Antananarivo. Spatial scanning was performed with a cylindrical window, moved in space and time for each geographical location (neighborhood) and size (limited to 50% of the study population). The cluster considered to be most likely is that for which the pattern is the least likely to be the same as that outside the window. Associated p-values for the likelihood were calculated to determine the significant differences. They are adjusted for the multiple testing stemming from the multitude of circles corresponding to different spatial locations and sizes of potential clusters evaluated [16]. Both the most likely clusters, and secondary clusters, were identified by Monte Carlo simulation.

The spatial model of the tuberculosis risk was mapped by using the Relative Risk (RR) for each neighborhood. It was calculated as the observed cases divided by the expected cases within the cluster divided by the observed cases divided by the expected cases outside the cluster [16]. ArcGIS Version 9.3 software was used to generate cartographic displays.

### Knowledge, Attitude and Practice (KAP) study

Two approaches were used to study tuberculosis-related KAP. The first approach was a quantitative survey of TB patients. The neighborhoods were randomly selected to constitute 3 groups of patients from neighborhoods with strong clustering, moderate clustering and without clusters. This was to include at least 50 TB patients. All the patients in the selected neighborhoods were visited by community health workers. The patients were questioned about their level of education and their knowledge of tuberculosis before they contracted the disease. The questions addressed knowledge of the symptoms of the disease, of its mode of transmission, the duration of treatment and free treatment. They were asked about their attitude towards relatives and family. No data were collected or sought for patients who had died or who had defaulted on treatment; other persons were not allowed to answer to the questions.

The second part of the KAP study was a qualitative survey in the healthy tuberculosis-free population, and involved the use of focus groups. It was conducted within the population of one neighborhood selected from each of the 3 types of neighborhoods (neighborhoods in the principal cluster, in the secondary cluster or outside the clusters). Three groups of patients were comprised from the patients for the KAP survey: patients from the principal cluster (group 1), those from the secondary cluster (group 2) and those from neighborhoods outside the clusters (group 3). The study sample was formed in close collaboration with the communal workers of the neighborhoods. The participants in the present study included only permanent residents of the Antananarivo Urban Township.

The sample for the qualitative survey was selected to provide a maximum of information rather than to be representative. The 3 neighborhoods chosen, according to the cluster gradient for this qualitative study were: Manarintsoa Isotry (principal cluster), Antsararay (secondary cluster) and Faliarivo Ambanidia (outside the cluster). Participants in the focus groups were recruited by home visit, and appointments were made to ensure their availability. Participation was voluntary, and the participants were not indemnified to favor free expression. For each neighborhood, 4 groups of 4 to 12 people were included in focus groups. The focus groups were each homogeneous for age and the gender: thus, one group was women aged from 15 to 24 years; one of women aged more than 24 years; one group of the men aged from 15 to 24 years; and one of the men that are aged more than 24 years. The aim of this homogeneity was to facilitate open discussions and to obtain points of view according to these categories.

Therefore, there were a total of 12 focus groups and additional information was collected by individual interviews of 9 people (3 people per zones from health community workers, neighborhood representatives or communal workers). They are the responsible for the health issues in their local communities especially that of Informing - Educating - Communicating these issues (IEC). They had voluntarily agreed to participate in the survey. A thematic guide composed of open and simple questions with common-language expressions was used to orient discussions about knowledge, attitudes and practices concerning tuberculosis. First, questions were about symptoms, treatment cost (with charge or free), mode of transmission and mean of diagnosis. Then there were questions about the people's perception of tuberculosis. They were asked about their outlook on tuberculosis in their relatives or friends. The sessions were conducted in the “Malagasy” language by a moderator and an observer who took notes during the group discussion. All discussions were recorded on a dictaphone.

Data for the KAP of tuberculosis cases was analyzed with R version 3.0.0 software (2013-04-03; copyright © 2013; the Statistical Computing Platform of the R foundation). Data were compared between clusters, and between clustered and non-clustered cases. Data from focus groups were studied by manual thematic analysis: this consisted of using a predefined analysis grid, composed of categories; the information was coded and manually processed.

### Ethical approval

This study received approval from the National Ethics Committee, and was authorized by the Ministry of Public Health in Madagascar (85-MINSANP/CE, September the 30^th^ 2011). The informed consent procedure included verbal explanation of the study objectives. Participants were encouraged to ask questions at any time. Participation in this study was voluntary. Written informed consent, in the local language, was obtained from all participants. This procedure was approved by National Ethics Committee.

All appropriate precautions were taken to preserve confidentiality, and anonymity of the collected data. Diffusion and the publication of the results strictly respect the measures forbidding access to information that is directly or indirectly nominative. Anonymity has been guaranteed.

## Results

### Detecting clusters of new cases of pulmonary tuberculosis

There were 4620 registered patients in the 18 DCT during the study period: 2225 of these patients were new pulmonary tuberculosis-positive cases. Their mean age was 43 years, and the sex ratio (male/female) was 1.45. Among tuberculosis pulmonary new cases, 1108 were resident in urban districts of Antananarivo.

The principal cluster was about 1.09 km across and covered 22 neighborhoods, mostly in the first district (18 neighborhoods) and partly in the fourth district (4 neighborhoods).

**Figure 1:**
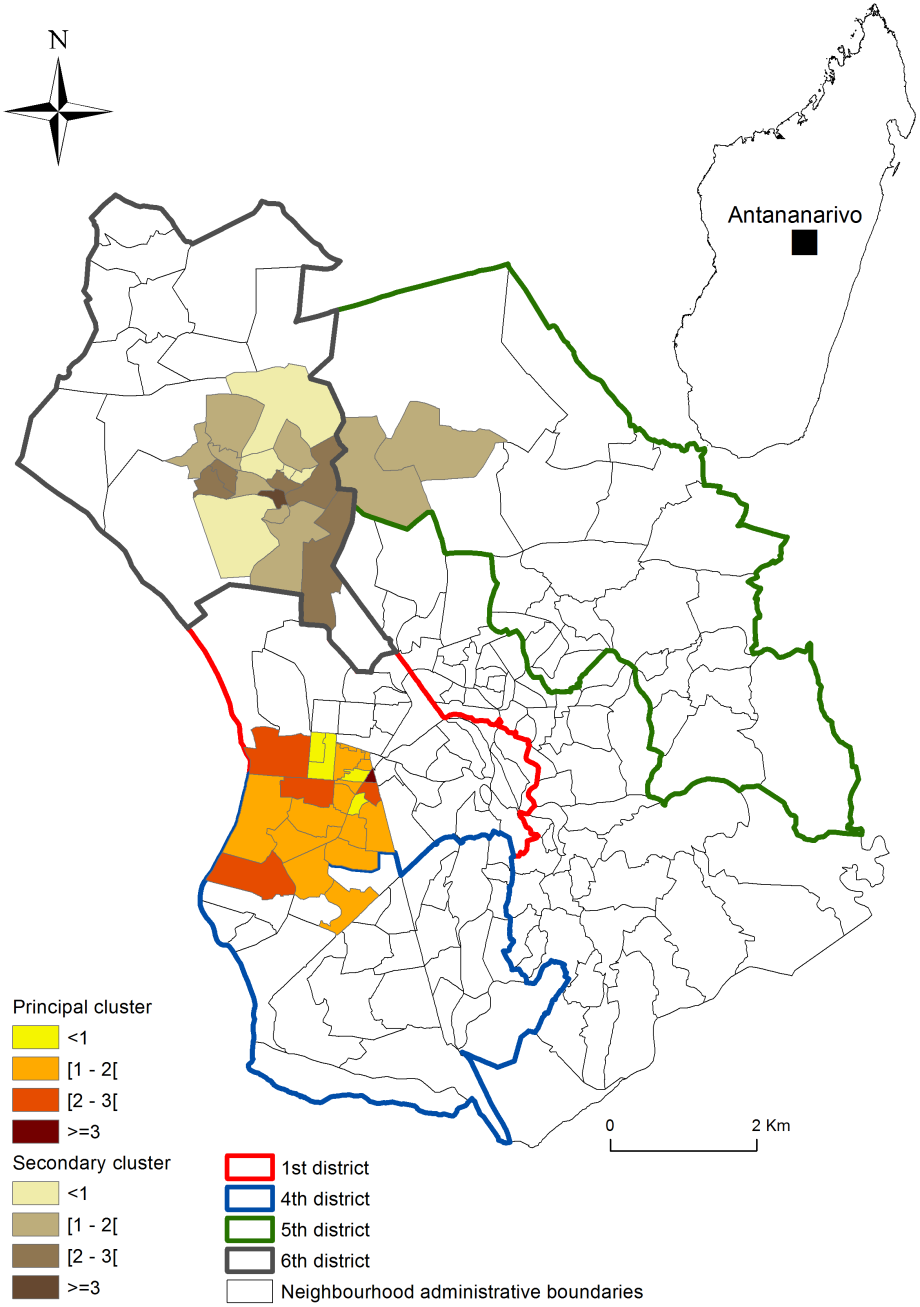
Relative risk of TB incidence, principal cluster was found in the 1^st^ and 4^th^ districts, secondary cluster was found in 5^th^ and 6^th^ districts.

A secondary cluster 1.77 km across was found, involving 18 neighborhoods in the sixth sub-district and 2 neighborhoods in the fifth (Table 1). The local relative risk for each neighborhood in the principal cluster was 0.57 to 4.72 and in the secondary cluster 0.6 to 3.71 (Figure 1).

**Table 1:** Description of cluster distribution, relative risk and number of neighborhoods included.

Cluster	N	Obs	Exp	RR	P-value
Most likely cluster	22	192	121.5	1.7	10^−6*^
Secondary cluster	20	116	71.2	1.6	10^−3*^

N: number of neighborhoods included in the cluster; Obs: number of observed new cases.

Exp: number of expected new cases; RR (Relative Risk): the estimated risk within the cluster divided by the estimated risk outside the cluster; *: Significant difference (P<0.05).

### KAP survey of new tuberculosis cases

Many patients in the neighborhoods included in the principal cluster could not be contacted (moving within, or away from, Antananarivo). Therefore, the number of patients randomly selected in the neighborhoods was increased to 83 of whom 28 agreed to participate in the survey. In the other neighborhoods, most patients refused to participate. Thus, only 20 of the 57 patients contacted in neighborhoods outside the clusters, and 32 of the 60 patients in the secondary cluster agreed to participate.

About 78.7% of the patients reported not knowing or having had contact with a TB case before their illness. This was most frequent in the zones belonging to the secondary cluster. Indeed, knowing a TB contact before their illness differed significantly between the 3 groups of patients (Fisher's exact test: p = 0.03). Of the investigated patients, 67% (53/79) informed their family or close friends about their illness. However, this notion of sharing of information about the illness differed according to the location of the patient (Fisher's exact test: p = 0.004). Patients from the zones outside the clusters were prone to share their health status compared to the patients inside the principal and secondary cluster. We counted 19 patients out of 53 (35.8%) from the zones outside the cluster, 17 (32.1%) from the principal cluster and 17 (32.1%) from the secondary cluster. Table 2 showed that most patients in the secondary cluster, *i-e* 15 out of 26 (57.7%), did not share information about their health status. Asked about their practices during treatment, 85% of the patients declared that they had not changed their attitude towards their illness. About 62.5% reported taking some special measures in their daily practice: 37.6% reported covering their mouth when coughing, 28% reported exposing the interior of the house to the sun and fresh air. Around 11.2% reported limiting visits to family or friends, and 4% stopped work during treatment. Some participants reported measures consisting in isolating the sick person (separate bedrooms) and separating their kitchenware and cutlery.

**Table 2:** Main results of KAP analysis and relation with cluster level; p-value indicates the significance of the clustering.

Cluster	Principal N(%)	SecondaryN(%)	No cluster	P-value
Education level				
- Elementary school	8(40)	7(35)	5(25)	NS*
- Secondary school	17(33.3)	23(45.1)	11(21.6)	
- University	3(33.3)	2(22.2)	4(44.4)	
Did you have knowledge about tuberculosis before treatment?				
- Yes	20(39.2)	20(39.2)	11(21.6)	NS
- No	8(27.6)	12(41.4)	9(31)	
Did you have contact with TB relatives or friends before treatment?				
- Yes	11(64.7)	2(11.8)	4(23.5)	P = 0.03**
- No	14(28.6)	22(44.9)	13(26.5)	
- Don′t know	3(21.4)	8(57.2)	3(21.4)	
When you knew that you had TB,				
Did you let others know it?				
- Yes	17(32.1)	17(32.1)	19(35.8)	P = 0.004**
- No	10(38.5)	15(57.7)	1(3.8)	
If yes, have you changed your attitude towards your circle or friends?				
- Yes	3(30)	3(30)	4(40)	NS
- No	24(35.3)	28(41.2)	16(23.5)	
- No response	1(50)	1(50)	0	

*Not Significant, P>0.05.

** Significant with the Fisher's Exact Test, P<0.05.

**Figure 2:**
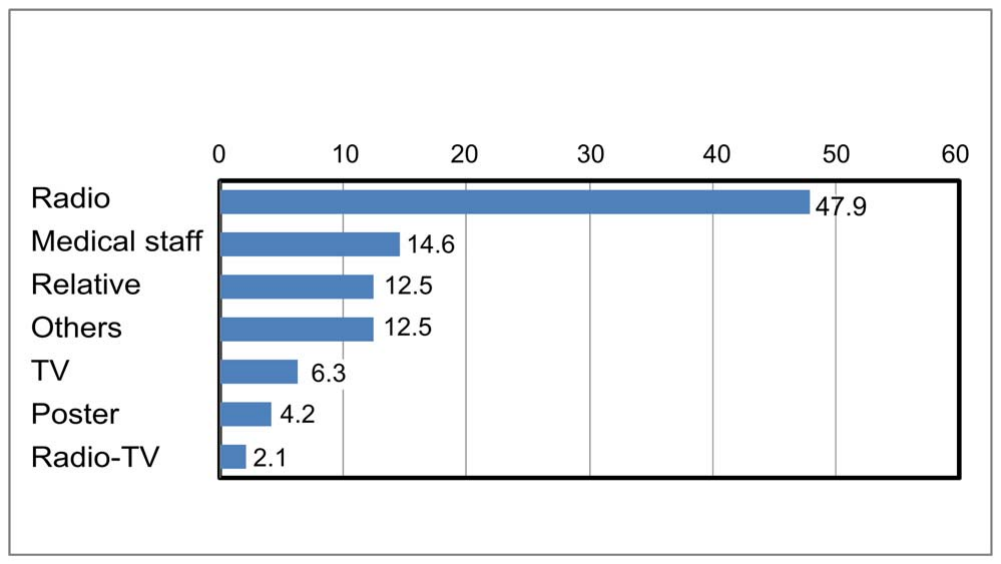
Sources of information; radio was the main source reported by TB patients, then the medical staff.

The most frequently reported source of information was the radio (Figure 2). The tuberculosis symptoms known by patients before their treatment were persistent cough and bloody sputum (65.8%), weight loss (16.6%) and asthenia (12.6%). Approximately 77.5% of TB patients reported that tuberculosis is contagious and most of the patients said that transmission is through droplets of saliva (from coughing or laughing) and sputum. Almost all the patients, 97.4%, believed that tuberculosis can be cured. Only 46.8% stated that before their TB diagnosis, they knew that treatment is free. Most, 87.5% had believed that the duration of treatment was 8 months.

### Tuberculosis-related KAP in the general population focus groups

A KAP survey was conducted in the tuberculosis-free general population by working with 12 focus groups including a total of 68 participants (mean age 34 years, range 15 to 80 years). The results of KAP analysis were aggregated by “*fokontany*”: group 1 for participants from the principal cluster, group 2 for participants from secondary cluster and group 3 for participants from those “*fokontany*” outside the two clusters.

The major sources of information were relatives with tuberculosis, and schools. Here are some examples of responses:

*“…Our neighbor when we lived in Ankorondrano, he had tuberculosis. This is what I know!”. “…We have already talked about it at school…”.*

Participants in group 1 believed that tuberculosis is a pulmonary disease. Participants in groups 2 and 3 stated that tuberculosis was defined as severe and chronic cough associated or not with other symptoms, like bloody sputum, breathing difficulties, fever and fatigue. Chronic cough was reported as the main symptom of tuberculosis by all three groups.

Participants affirmed that tuberculosis is not contagious. Group 1 and group 2 evoked “dirtiness” as the cause of tuberculosis: for example, contaminated foods, unpasteurized milk, contaminated water and air, and poor bodily hygiene.

Participants of groups 1 and 2 said that there are no risk factors for tuberculosis; everybody is susceptible to catching the disease. Participants in groups 2 and 3 mentioned risk factors, such as poverty, promiscuity, contact with tuberculosis patients, and the lack of vaccination especially for children. Poor diet, alcoholism, smoking and hard work were cited as risk factors. Group 3 added immunodeficiency.

*“…There is no risk factor: who is affected is affected…”*

*“…People vulnerable to tuberculosis are those who drink alcohol, who smoke cigarettes, do not eat enough calcium and vitamins and do not drink enough water…”*

For the participants of all three groups, tuberculosis is curable if treatment and care are started early; otherwise it can lead to death. For example:

*“… Yes, it is curable if one consults a doctor early, but do not resort to self-medication…”.*

*“… Curable if the patient is not ashamed to see a doctor, if he follows the doctor's recommendations and takes his medication regularly as well…”*

*“… The disease is fatal if not treated correctly…”*

Twenty of the 47 participants in groups 2 and 3 did not know anything about the treatment of tuberculosis. Participants said that the treatment duration depends on the symptoms of the illness and the financial condition of the patients; they described durations ranging from 4 days to many years. Participants affirmed that treatment and care are free in public health centers, but not in private health centers.

For example:

*“…The treatment lasts two, three or four months… according to the financial condition. You have money; you stay. If you do not have any, you go back home…”.* Participants did not believe that tuberculosis is an illness of the poor. According to them, tuberculosis can affect everyone. The majority of participants in group 2 and group 3 felt that tuberculosis is associated with shame; there is a stigma attached to tuberculosis.

*“…Tuberculosis is painful and shameful as cracks at the heels …”.*

*“One doesn't reveal it, one immediately takes the patient to the physician because it is dishonoring”.*

Participants, particularly in groups 2 and 3, had positive attitudes towards tuberculosis patients. However, group 1 indicated that patients' kitchen utensils should be kept separated, and the patients themselves isolated; they stated taking precautions, including restricting conversations with patients, and washing hands after visits. Participants in groups 2 and 3 felt obliged to restrict contact to phone calls. Some participants of group 1 would visit tuberculosis patients if the patients were taking appropriate treatment.

*“…The relationship doesn′t change, but one moves away a little…, because it is a contagious disease…”.*

*“…If the illness is very serious, I would worry about visiting someone with tuberculosis…”.*

*“As far as I know, one can visit people with tuberculosis, but not get too close to them…”.*

## Discussion

Socio-economic status is known as a vulnerability factor of TB but our study assesses the contribution of KAP to the case distribution pattern. Our results show that the spatial aggregation zones for tuberculosis in the Antananarivo Urban Township have not changed substantially since the previous survey [10], [17]. TB cases still tend to aggregate primarily in clusters in the 1st and 4th district; the secondary clusters are in the 5th and the 6th district. These districts are among the most densely populated in the Antananarivo Urban Township. Our main finding was about patients' and population's attitude towards tuberculosis. We found that patients do not pass on information about being ill, mostly in the secondary cluster. This is consistent with the fact that a large number of TB cases did not know or had not known if they were in contact with TB patients before contracting the disease. Attitudes described by the population in the qualitative survey were consistent with this finding. The tendency to hide the disease was also mentioned by the focus group. Withholding information on having contracted the disease is most likely the reason for the spatial clustering of TB. Likewise, patients have not changed their attitude towards the people around them. In addition, a patient who hides his condition does not benefit moral and financial support of his family. These patients present a greater risk of not benefiting appropriate care and help to spread the disease. It confirms that the stigma linked to the disease still dictates the behavior of patients. This may constitute an obstacle to the investigation of TB contact, and consequently in the success of the program.

The absence of a cluster does not mean that the transmission risk is zero. Nevertheless, mathematical analysis using a Bayesian approach combined with generalized linear mixed model showed a significantly lower risk in neighborhoods in locations not detected by spatial scan statistics [17]. A long socio-political crisis was experienced by the country; this cluster stability seems relative. The population was weakened by the crisis which may have increased the transition to clinical forms of latent TB. The recommended strategy by the National Program of Tuberculosis Control is based on passive detection of cases. The stability of the cluster extent shows that such passive detection is not enough to clean the transmission foci or to improve the situation in the areas where the risk is very high. Reorienting strategy about TB detection in active investigation of close contacts of persons with infectious tuberculosis, and active case research in the community is needed.

In addition, we found that the ignorance of the basic knowledge and the free treatment about TB may be the cause of the insidious spread of this disease. TB patients evoked persistent cough and bloody sputum as the main symptoms, and these were also identified by most of the tuberculosis-free participants. However, wrong information persists about tuberculosis transmission, e.g. transmission through genital route, by dust, by food and by contaminated clothing was cited. Knowledge about treatment is still limited and only 46.8% of the patients knew, before TB diagnosis, that treatment is free. The presentation of the drugs is even less known. This lack of knowledge about treatment is undoubtedly a disincentive to be consulted diagnosed and then cared while the weakness and the loss of prosperity reduced the ability of the patients to afford access to basic health care. Qualitative study did not show any relevant difference of KAP between the groups, but it should be an indicator of the general population's knowledge. The KAP survey in general population confirms that knowledge of treatment is poor. Participants reported that duration of treatment depends on money and on the financial condition of the patient (from four days to many years). Consequently, information campaigns and dissemination need to be improved; patients must be incited to come forward for diagnosis, and to be encouraged to complete their treatment within the appropriate time.

These results show that IEC programs should be reinforced. The existence of Community Health Workers is an asset in the IEC health system, but they need more appropriate training on how to approach the patients and to give them information. Their knowledge is not much more than that of the population in general, which also needs edification.

Randremanana *et al.* [10], [17], found a strong principal cluster in the 1^st^ and 4^th^ districts for the period of August 2004-July 2006. A TB case survey in an urban West African setting, using the same tools and approach, showed evidence of clustering in Greater Banjul, the Gambia. A study conducted in Dabat, Ethiopia, also had shown a significant concentration of clusters involving permanent residents in certain locations [18]. These previous studies confirm the value of Geographic Information Systems and spatial scan statistics for identifying clusters and focal transmission of TB in communities.

Touray *et al*. suggested that community-based active case detection would confirm the existence of clusters [19]. Morrison *et al.* had reported that latent tuberculosis infection was found in 51.4% of contact investigations [20]. Gonzàlez-Ochoa *et al.* found that tracking cases in populations at risk increases rates of case detection [21]. These authors thus illustrate the importance of active detection of tuberculosis by investigating patient contacts. Advanced IEC strategies, recommended by the National Tuberculosis Program, are used by community health workers (CHW) to track people with chronic coughs and family contacts at risk of tuberculosis transmission. The door-to-door method has also been used, but tuberculosis detection remains passive. The persistence of these clusters suggests that case detection in these zones at risks in Antananarivo Urban Township should be fruitful. Thus, the efforts of medical staff to educate patients may have a significant impact on their knowledge but little evidence has been noticeable on their attitude change.

Vukovic *et al.* found that personal contact with doctors to get information is appreciated [22]. In Ethiopia, the majority of interviewees reported persistent cough as the main symptom of TB (74.3%) [23]. The “evil eye” was commonly cited as a cause of TB in Ethiopia. In Tanzania, only 30% of the study population had an adequate knowledge about TB [24]. In Pakistan, a cross-sectional survey in the general population had shown that participants knew about correct treatment but less than half were aware that diagnosis and treatment are free [25]. One of the limitations of our study is the risk of confusion of knowledge before and after diagnosis: the survey of tuberculosis patients was retrospective, so the issue is difficult to assess.

A survey in Ethiopia showed that 69% of patients have the impression of being rejected by the community, and 78.3% of the population fear contact with TB patients [26]. In rural southwest Ethiopia, 51.2% of TB patients reported stigmatization [27]. A population study by Deribew showed that 56% of respondents had a high prejudice towards TB patients [28]. Singh *et al.* (2002) found that discrimination remains substantial in Lok Nayak Colony, Delhi, and includes isolating TB patients (71%), avoiding sharing food (74.1%), and other behaviors [29]. However, as found in our focus groups, having a close friend or relative suffering of TB can lead to individuals being better informed about the disease.

Tuberculosis is characterized by human-to-human transmission. Consequently, the spatial distribution of the disease and the geographic aggregation of cases are determined by human behavior. This informative KAP report investigation can be considered as a basis of further investigations, particularly to assess the impact of information programs, on perception and experience of tuberculosis. This survey was conducted in Antananarivo where access to information, education and communication has improved; however, it is also important to assess the impact of the IEC programs for tuberculosis in the remote regions. Nevertheless, this study contributes to identify community gaps in the knowledge of tuberculosis. This type of survey could be repeated in other zones where the tuberculosis-related KAP of TB patients, and the general population is less well-known and not documented; the findings could be used to adapt control to the local particularities. This may help improve the success and development of the National Tuberculosis Program.
